# Contralateral surgical exploration during inguinal hernia repair in infants (HERNIIA trial): study protocol for a multi-centre, randomised controlled trial

**DOI:** 10.1186/s13063-021-05606-w

**Published:** 2021-09-30

**Authors:** Kelly M. A. Dreuning, Maurits W. van Tulder, Jasper V. Been, Maroeska M. Rovers, Jurgen C. de Graaff, Markus F. Stevens, Johannes R. Anema, Jos W. R. Twisk, L. W. Ernest van Heurn, Joep P. M. Derikx, on behalf of the HERNIIA study group

**Affiliations:** 1 Department of Paediatric Surgery, Emma Children’s Hospital, Amsterdam UMC, University of Amsterdam & Vrije Universiteit Amsterdam, Amsterdam Reproduction and Development Research Institute and the Amsterdam Public Health Research Institute, P.O. Box 22660, 1100 DD Amsterdam, The Netherlands; 2 Department of Health Sciences and Amsterdam Movement Science research institute, Faculty of Sciences, Vrije Universiteit Amsterdam, Amsterdam, The Netherlands; 3 Department of Physiotherapy & Occupational Therapy, Aarhus University Hospital, Aarhus, Denmark; 4 Division of Neonatology, Department of Paediatrics, Sophia Children’s Hospital, Erasmus University Medical Centre, Rotterdam, The Netherlands; 5 Department of Public Health, Erasmus MC, University Medical Centre Rotterdam, Rotterdam, The Netherlands; 6 Department of Obstetrics and Gynaecology, Sophia Children’s Hospital, Erasmus University Medical Centre, Rotterdam, The Netherlands; 7 Radboud Institute for Health Sciences, Department of Health Evidence, Radboud University Medical Centre, Nijmegen, The Netherlands; 8 Department of Anaesthesiology, Erasmus MC - Sophia Children’s Hospital, Rotterdam, The Netherlands; 9 Department of Anaesthesiology, Amsterdam UMC, University of Amsterdam, Amsterdam, The Netherlands; 10 Department of Public and Occupational Health, and the Amsterdam Public Health Research Institute, Amsterdam UMC, Vrije Universiteit Amsterdam, Amsterdam, The Netherlands; 11 Department of Methodology and Applied Biostatistics, and the Amsterdam Public Health research institute, Vrije Universiteit, Amsterdam, The Netherlands

**Keywords:** Inguinal hernia, Inguinal hernia repair, Metachronous hernia, Contralateral exploration, Cost-effectiveness, Infants

## Abstract

**Background:**

The incidence of metachronous contralateral inguinal hernia (MCIH) is high in infants with an inguinal hernia (5–30%), with the highest risk in infants aged 6 months or younger. MCIH is associated with the risk of incarceration and necessitates a second operation. This might be avoided by contralateral exploration during primary surgery. However, contralateral exploration may be unnecessary, leads to additional operating time and costs and may result in additional complications of surgery and anaesthesia. Thus, there is no consensus whether contralateral exploration should be performed routinely.

**Methods:**

The Hernia-Exploration-oR-Not-In-Infants-Analysis (HERNIIA) study is a multicentre randomised controlled trial with an economic evaluation alongside to study the (cost-)effectiveness of contralateral exploration during unilateral hernia repair. Infants aged 6 months or younger who need to undergo primary unilateral hernia repair will be randomised to contralateral exploration or no contralateral exploration (*n* = 378 patients). Primary endpoint is the proportion of infants that need to undergo a second operation related to inguinal hernia within 1 year after primary repair. Secondary endpoints include (a) total duration of operation(s) (including anaesthesia time) and hospital admission(s); (b) complications of anaesthesia and surgery; and (c) participants’ health-related quality of life and distress and anxiety of their families, all assessed within 1 year after primary hernia repair. Statistical testing will be performed two-sided with *α* = .05 and according to the intention-to-treat principle. Logistic regression analysis will be performed adjusted for centre and possible confounders. The economic evaluation will be performed from a societal perspective and all relevant costs will be measured, valued and analysed.

**Discussion:**

This study evaluates the effectiveness and cost-effectiveness of contralateral surgical exploration during unilateral inguinal hernia repair in children younger than 6 months with a unilateral inguinal hernia.

**Trial registration:**

ClinicalTrials.govNCT03623893. Registered on August 9, 2018

Netherlands Trial Register NL7194. Registered on July 24, 2018

Central Committee on Research Involving Human Subjects (CCMO) NL59817.029.18. Registered on July 3, 2018

## Administrative information

The order of the items has been modified to group similar items (see http://www.equator-network.org/reporting-guidelines/spirit-2013-statement-defining-standard-protocol-items-for-clinical-trials/).

**Table:** 

Title {1}	Contralateral surgical exploration during inguinal hernia repair in infants (HERNIIA trial): Study protocol for a multi-centre, randomised controlled trial.
Trial registration {2a and 2b}.	NCT03623893 [clinicalTrials.gov][registered on August 9, 2018] https://clinicaltrials.gov/ct2/show/NCT03623893 NL7194 [Netherlands Trial Register][registered on July 24, 2018] https://www.trialregister.nl/trial/7194 NL59817.029.18 [Registry ID: CCMO][registered on July 3, 2018]
Protocol version {3}	Version 1.8, date 30-06-2020
Funding {4}	This work is funded by The Netherlands Organisation for Health Research and Development (ZonMw), grant number 852001903.
Author details {5a}	Kelly MA Dreuning: Emma Children’s Hospital, Amsterdam UMC, The Netherlands. Maurits W van Tulder: Vrije Universiteit Amsterdam, The Netherlands; Aarhus University Hospital, Denmark. Jasper V Been: Sophia Children’s Hospital, Erasmus University Medical Centre Rotterdam, The Netherlands. Maroeska M. Rovers: Radboud University Medical Centre Nijmegen, The Netherlands. Jurgen C de Graaff: Sophia Children’s Hospital, Erasmus University Medical Centre Rotterdam, The Netherlands. Markus F Stevens: Amsterdam UMC, University of Amsterdam, The Netherlands. Johannes R Anema: Amsterdam UMC, Vrije Universiteit Amsterdam, Amsterdam, The Netherlands. Jos WR Twisk: Vrije Universiteit, Amsterdam, The Netherlands. LW Ernest van Heurn: Emma Children’s Hospital, Amsterdam UMC, The Netherlands Joep PM Derikx: Emma Children’s Hospital, Amsterdam UMC, The Netherlands.
Name and contact information for the trial sponsor {5b}	Investigator initiated clinical trial; L.W. Ernest van Heurn (Principal Investigator) e.vanheurn@amsterdamumc.nl
Role of sponsor {5c}	This is an investigator initiated clinical trial. Therefore, the funder (ZonMw) plays no role in the design of the study and collection, analysis, and interpretation of data and in writing the manuscript.

## Introduction

### Background and rationale {6a}

Inguinal hernia is the most common paediatric surgical disorder with an incidence of 0.8–5% during childhood age and up to 30% in infants born preterm [1, 2]. The processus vaginalis normally obliterates spontaneously before or shortly after birth. If it remains patent, then fluid, fat or intestines can move into the open inguinal canal and present as a clinically visible hernia. Surgical repair (i.e. closing the patent processus vaginalis (PPV)) is recommended shortly after diagnosis because of the risk of incarceration, which is reported to be 3–30% in the first 6 months of life and even higher if the infant was born preterm [3].

Eighty percent of children with inguinal hernia present with a unilateral hernia, of which 10–15% develops a metachronous contralateral inguinal hernia (MCIH) [4]. Infants younger than 6 months old have the highest risk of developing MCIH [3, 5–7]: the overall risk for MCIH in 49,568 children undergoing unilateral hernia repair from 61 studies was 5.8%. However, in infants who were younger than 6 months (*n* = 1470), the risk for MCIH development was substantially higher at 12.4% [4]. Because an MCIH almost invariably necessitates a second operation and increases the child’s potentially harmful exposure to a second anaesthesia session, preventive strategies (e.g. contralateral exploration) have been proposed since the 1950s. Exploration of the asymptomatic, contralateral groin during unilateral hernia repair enables simultaneous inspection of the contralateral processus vaginalis and subsequent closing of a PPV, if present. Consequently, repair of an open processus vaginalis during contralateral exploration can prevent potential MCIH [8]. Especially since the predictive value of prognostic factors [9] and diagnostic modalities (e.g. preoperative ultrasonography) for development of MCIH are insufficient to specifically target children that will develop MCIH [10], routine contralateral exploration might therefore be beneficial. In infants less than 6 months old, nine contralateral explorations will be required to prevent one MCIH [4, 11].

Surgeons may prefer performing contralateral exploration because of high MCIH incidence, high incarceration rate and repeated anaesthesia, carrying risk for near critical incidents (apnoea risk up to 12%) [12]. The US Food and Drug Administration (FDA) recently released an official warning regarding the potentially harmful impact of repeated general anaesthesia on the child’s brain [13]. Indeed, previous studies have shown that multiple exposures to general anaesthesia before 3 years of age is associated with neurocognitive and developmental problems (e.g. learning disabilities) [14–17]. More recent research has shown that the potential impact of a single session of general anaesthesia for hernia repair on functional brain development is not detectable [18, 19]. Yet, the effect of repeated anaesthesia on brain development remains unclear [13, 18]. These findings highlight the importance of preventing repeated surgery and anaesthesia. Contralateral exploration may help to reduce the frequency and total length of anaesthesia in children that develop MCIH after unilateral hernia repair.

Nonetheless, contralateral exploration also carries additional risks of operative complications: wound infection occurs in 0.6–1.2% of the children, hematoma in 0.1%, testicular atrophy in 0–0.3% and ipsilateral recurrence in 0.4–1.2% [20, 21]. In preterm babies, these risks are even higher: the recurrence rate varies from 2 to 8.6% and the risk of testicular atrophy, which may be accompanied by loss of function, ranges from 2 to 30% [3, 5]. If both testes are affected, boys may become infertile. As not all PPVs necessarily become hernias, contralateral exploration and its additional risks will be unnecessary in some children [22]. Consequently, the debate continues whether or not to perform contralateral inguinal exploration in infants with inguinal hernia [4, 9, 20]. Cost-effectiveness of both strategies in children has never been assessed.

### Objectives {7}

We hypothesise that open contralateral exploration with subsequent contralateral hernia repair prevents second surgery and repeated exposure to anaesthesia and that the risk of surgical complications equals the complication rate of symptomatic hernia repair. Prevention of MCIH and avoiding second anaesthesia and surgery together with its potential risks and complications possibly outweighs the potential burden of contralateral exploration. Complications following negative contralateral exploration will be even less.

### Study objectives

The Hernia Exploration oR Not In Infants Analysis (HERNIIA) trial aims to study the effectiveness and cost-effectiveness of contralateral inguinal exploration in infants aged 6 months or younger with a unilateral inguinal hernia compared with unilateral repair only.

### Trial design {8}

A prospective, parallel group, multicentre randomised controlled trial (RCT) will be conducted including infants aged less than 6 months who present with unilateral inguinal hernia. The SPIRIT 2013 Statement (Standard Protocol Items: Recommendations for Interventional Trials) and SPIRIT 2013 Explanation and Elaboration paper for Randomised Trials [23] were used for complete and transparent reporting of the trial protocol.

## Methods: participants, interventions and outcomes

### Study setting {9}

Five paediatric surgical centres in The Netherlands (Amsterdam UMC: Emma Children’s Hospital AMC and VU medical centre; University Medical Centre Groningen; Maastricht University Medical Centre; Erasmus MC - Sophia Children’s Hospital Rotterdam), Juliana Children’s Hospital Den Haag and Maxima Medical Centre Veldhoven participate in this trial. Inguinal hernia repair is performed in clinical or day care setting depending on the postnatal age and gestational age of the infant.

### Eligibility criteria {10}

#### Inclusion criteria

Infants aged younger than 6 months at first presentation with a primary unilateral inguinal hernia undergoing open hernia repair are considered eligible for inclusion.

#### Exclusion criteria

Infants with (1) incarcerated inguinal hernia requiring urgent surgery, (2) a ventricular-peritoneal drain and (3) non-descended testis will be excluded.

### Who will take informed consent? {26a}

The surgeon explains the study to the parents/caretakers, hands over the information letter and informed consent form, and asks permission to hand over their contact details to the research team. A member of the research team will contact the parents/caretakers to provide more details and answer questions if necessary. As most infants are operated within 3–4 weeks after the outpatient clinic visit, there will be enough time for parents/caretakers to discuss the study and ask any questions before consent needs to be given. At each centre, all (fellow) surgeons who will be involved in this clinical trial will receive training in order to provide information to the parents/caretakers according to the standard operating procedure (SOP) and Good Clinical Practice (GCP) guidelines.

### Additional consent provisions for collection and use of participant data and biological specimens {26b}

Not applicable, as these will not be collected.

## Interventions

### Intervention description {11a}

Open inguinal hernia repair in infants younger than 6 months old is performed by experienced (fellow) paediatric surgeons. It is the most commonly performed operation in children and no substantial variations in operation technique exist. Therefore, no additional training is necessary for the purpose of this study.

#### Intervention group: unilateral hernia repair with contralateral exploration

Surgical inguinal hernia repair is done similarly in all patients. An incision is made in the groin. The hernia sac is identified, divided from the vas deferens and testicular vessels (in boys) and cleaned to the level of the internal inguinal ring. It is twisted on itself and ligated at the level of the internal inguinal ring. The skin is closed. Duration of unilateral inguinal hernia repair is approximately 60 min (including anaesthesia time). Exploration of the contralateral side and potential contralateral hernia repair will increase anaesthesia time by approximately 15 min.

Contralateral exploration will be used to identify a PPV or hernia on the other side than the side on which the child has to be operated on. If during contralateral exploration, a hernia or PPV is present, contralateral repair will be performed. This will be exactly the same procedure as the inguinal hernia repair on the symptomatic side. If a hernia or PPV is absent during contralateral exploration, the skin will be closed. There is no need for extension of postoperative hospital admission or addition of other interventions.

### Explanation for the choice of comparators {6b}

#### Control group: unilateral hernia repair without contralateral exploration

In participants who are allocated to the control group, only unilateral hernia repair (without contralateral exploration) will be performed.

### Criteria for discontinuing or modifying allocated interventions {11b}

Parents can decide to leave the study at any time for any reason, and without any consequences. The investigator can also decide to withdraw a subject from the study for urgent medical reasons. Participants will not be replaced after withdrawal. Protocol violations (e.g. if study participants do not receive the treatment strategy which is allocated by randomisation) will be recorded.

### Strategies to improve adherence to interventions {11c}

Not applicable, since the intervention in both groups comprises surgical treatment.

### Relevant concomitant care permitted or prohibited during the trial {11d}

Concomitant surgery such as orchidopexy is permitted during the trial, but will be registered.

### Provisions for post-trial care {30}

The sponsor has an insurance, which is in accordance with the legal requirements in the Netherlands (Article 7 WMO (Medical Research Involving Human Subjects Act)). This insurance (Onderlinge Waarborgmaatschappij Centramed B.A. PO Box 7374, 2701 AJ Zoetermeer) provides cover for damage to research subjects through injury or death caused by the study. The insurance applies to the damage that becomes apparent during the study or within 4 years after the end of the study. Subjects who participate in the study will receive information about this insurance.

## Outcomes {12}

### Primary outcome measure

Primary outcome is the proportion of reoperations within 1 year after primary hernia repair. This will be calculated using the number of infants that undergo a second operation related to inguinal hernia repair within 1 year following primary repair, as a fraction of the total number of infants in that group. All participants are seen by a (fellow) paediatric surgeon at the outpatient clinic or via a digital consult 1 year after primary hernia repair. The latter is done to score development of contralateral hernia, testicular atrophy and/or recurrence.

### Secondary outcome measures

#### Total duration of operation(s) including anaesthesia time and hospital admission(s)

Duration of operation(s) including anaesthesia time will be calculated in minutes from start of anaesthesia induction until end of anaesthesia. Length of hospital stay will be calculated as the number of days a patient is admitted to the hospital. This will be recorded by the (fellow) paediatric surgeon during each separate hospital admission/visit for inguinal hernia (repair).

#### Complications of anaesthesia and surgery

Complications related to inguinal hernia within 1 year after primary repair are as follows: wound infection, hematoma, hydrocele, testicular atrophy, apnoea or inguinal hernia recurrence. Apnoea, wound infection, hematoma and hydrocele will be assessed during hospital admission via monitoring (if applicable) and by the (fellow) paediatric surgeon. After hospital discharge, these complications will be assessed through a phone call performed by one of the investigators 4 weeks following surgery. Testicular atrophy and inguinal hernia recurrence will be assessed at the outpatient clinic 1 year following surgery. If there is partial or complete testicular atrophy of one testis (i.e. one of the testes is smaller compared to the other at follow-up), additional ultrasonography of both testes will be performed. Complications are defined as:

- Testicular atrophy: No palpable testicular tissue in scrotum (=complete testicular atrophy) or testis documented to be smaller at follow-up than at time of inguinal hernia repair (=partial testicular atrophy) at operated side(s).
- Wound infection/surgical site infection: Infection occurring within 30 days of surgery; infection involving only the skin and subcutaneous tissue; at least one of the following: (a) purulent discharge from a superficial infection or (b) organisms isolated from aseptically obtained wound culture; and at least one of the following signs of infection: (a) pain or tenderness, (b) localised swelling and (c) redness or hyperthermia [24].
- Apnoea: Apnoea of infancy is defined as “an unexplained episode of cessation of breathing for 20 seconds or longer, or a shorter respiratory pause associated with bradycardia, cyanosis, pallor, and/or marked hypotonia requiring intervention”. Types of interventions recorded will include stimulation, assisted ventilation, continuous positive airway pressure (CPAP), endo-tracheal intubation and administration of methylxanthine [25].
- Recurrence: the hernia returns at the previously operated side and a reoperation on the same side is warranted.

#### Health-related quality of life (HRQOL) and parental distress and anxiety

HRQOL of the operated infants, parental distress and parental anxiety will be measured by three different validated questionnaires next to the socio-demographic list. HRQOL of the operated infants will be calculated using the TAPQOL (TNO-AZL Preschool Children Quality of Life) [26] scores, with higher scores (range 0–100) indicating better HRQOL. Parental distress and anxiety levels will be calculated using the State-Trait Anxiety Inventory (STAI) [27] and the Distress Thermometer for Parents (DT-P) [28], indicating greater distress and anxiety when revealing higher scores. The questionnaires will be completed by the parents/caretakers at baseline before surgery, 4 weeks and 1 year after primary hernia repair and, if relevant, prior to and 4 weeks after re-operation for MCIH.

## Participant timeline {13}

### Baseline assessment

**Fig. 1:**
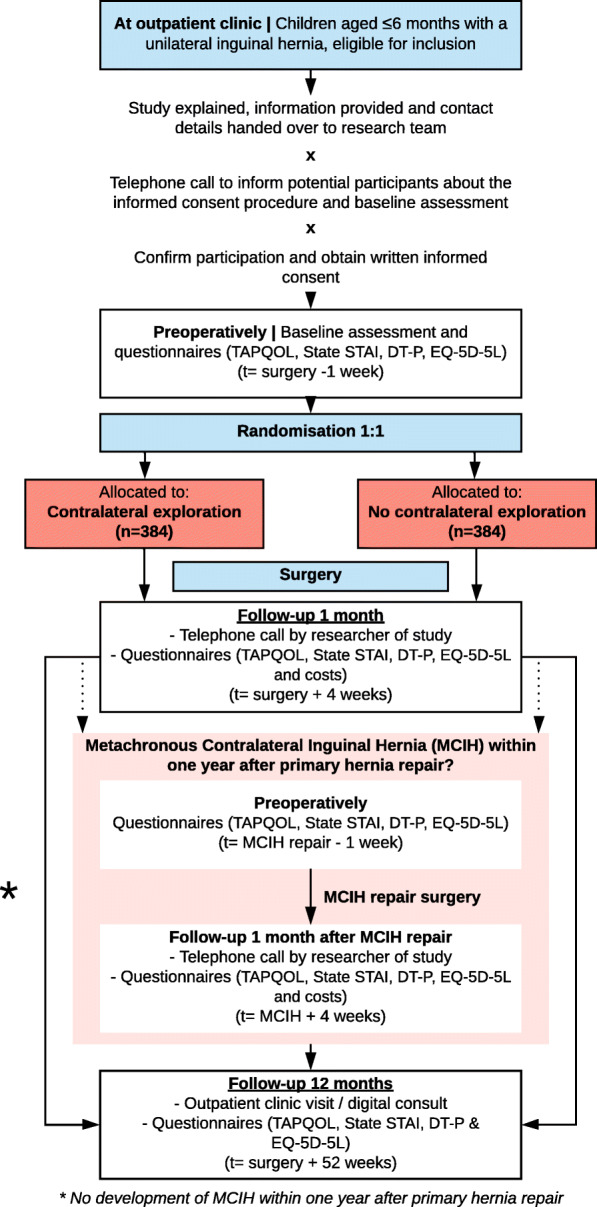
Flow chart of participants included in the study

After enrolment of participants in the study, demographics and baseline characteristics including sex, date of birth, medical history, gestational age, birth weight, side of the inguinal hernia and other hernia characteristics (e.g. history of incarceration) will be collected. Also, parents/caretakers receive four questionnaires (TAPQOL, State STAI, the DT-P and EQ-5D-5L (EuroQol 5D) that we ask them to complete before the intervention (Fig. 1).

### Post intervention assessment

Primary and secondary outcomes will be assessed during hospital admission, 4 weeks after primary hernia repair and 1 year after primary hernia repair. If patients develop MCIH within 1 year after primary inguinal hernia repair, baseline and outcome assessments will also be performed during and 4 weeks after MCIH repair.

## Sample size {14}

Data from the latest systematic review shows that the incidence of infants younger than 6 months of age who develop MCIH is 12.4% [4]. Retrospective data from the Emma Children’s Hospital AMC, where in 2000/2001 contralateral exploration was routinely performed, shows similar results: 8/92 patients did not undergo contralateral exploration and subsequently one patient (12.5%) developed MCIH. Eighty-four patients underwent unilateral hernia repair with contralateral exploration of which one patient (1.2%) developed MCIH.

The expected percentage of children requiring one or more reoperations because of MCIH in the first year following hernia repair is approximately 10%. We consider a reduction of children requiring reoperation from 10 to 2.5% as clinically relevant. A total sample size of 378 patients is needed to detect such a reduction with a power of 0.80 at a two-sided alpha of 0.05 (nQuery Advisor 7.0) [29]. Taking into account 10% loss to follow-up, we need to include 416 children.

## Recruitment {15}

Recruitment of participants started at 17 April 2019 and will be continued until the required number of participants will be enrolled. Infants with a unilateral inguinal hernia will be identified by the (fellow) paediatric surgeon at the outpatient clinic or neonatal intensive care unit (NICU) of the participating centres (Fig. 1). Currently (dd. August 24th 2021), 302 patients are included in this study. To enhance patient participation in this trial, one researcher coordinates the potential inclusions in all participating centres and strives to limit the number of eligible patients that were missed for inclusion. Over the past year, there has been some delay due to COVID-19; however, we expect to complete the recruitment of patients in 6 to 12 months.

## Assignment of interventions: allocation

### Sequence generation {16a}

Study participants will be randomly assigned (1:1) to the intervention (contralateral exploration) or control group (no contralateral exploration) using Castor Electronic Data Capture (Castor EDC), a web-based electronic case record form and randomisation program that is compatible with the GCP guidelines [30].

### Concealment mechanism {16b}

Treatment allocation will be concealed by using a web-based application with a computer-generated list with varying block sizes that will not be disclosed and is stratified by centre. Participants and care providers are aware of allocation.

### Implementation {16c}

After obtaining informed consent, participants will be randomly allocated to one of the two study groups by the researcher using Castor EDC.

## Assignment of interventions: blinding

### Who will be blinded {17a}

Blinding of patients, parents or caregivers is impossible because of the nature of the intervention since a scar will develop and instructions have to be given for the postoperative treatment of the wound(s). Outcome assessors and statistician blinded to study groups will conduct all analysis.

### Procedure for unblinding if needed {17b}

This is an open label trial; therefore, an unblinding procedure does not apply.

## Data collection and management

### Plans for assessment and collection of outcomes {18a}

Data of participants and findings for primary and secondary outcome measures will be recorded by the (fellow) paediatric surgeon or one of the investigators in a web-based electronic case record form using Castor EDC [30]. Parents/caretakers will use the online KLIK PROM (patient-reported outcome measures) portal (www.hetklikt.nu) to answer questionnaires.

The study will collect the following baseline characteristics and data from the participants: sex, gestational age, birth weight, comorbidities, inguinal hernia in first degree siblings, age and weight at day of surgery, preoperative size of both testes (in boys), side of the inguinal hernia and other hernia characteristics (e.g. preoperative history of hernia incarceration and surgical findings during contralateral exploration (i.e. presence of a PPV or not), total duration of surgery and hospital admission, total number of hospital visits and complications.

#### Health-related quality of life (HRQOL) and parental distress and anxiety

HRQOL of the operated infant will be assessed using the TAPQOL (TNO-AZL Preschool Children Quality of Life), a validated parent-reported questionnaire that is clustered into 12 multi-item scales in which questions are answered on a 3-point Likert scale, with higher scores (range 0–100) indicating better HRQOL [26]. Parental distress and anxiety levels will be measured with the State-Trait Anxiety Inventory (STAI) [27], a validated questionnaire that measures State Anxiety on a 4-point scale indicating greater anxiety when revealing higher scores. Last, parental distress will be measured using a brief version of the Distress Thermometer for Parents (DT-P), including the thermometer on which parents rate their distress (from 0, no distress, to 10, extreme distress) and a problem list to identify sources of distress in two domains: emotional and parenting [28]. The DT-P is a well-validated, brief screening instrument that is frequently used in Dutch clinical practice as quick screening to identify distress and everyday problems in parents of children who need medical treatment.

### Plans to promote participant retention and complete follow-up {18b}

Once a patient is enrolled and randomised, the researchers will make every reasonable effort to follow the study participant for the entire study period. Questionnaires (and if necessary reminders for completion) are sent using an online portal, thereby enabling parents to complete them at any convenient moment. Patients withdrawn from treatment will be asked to visit the outpatient clinic 1 year after surgery to investigate the primary endpoint of the study.

### Data management {19}

The trial will be conducted according to the study protocol and in compliance with the GCP guideline, to provide assurance that the rights, safety and well-being of trial subjects are protected and that the trial data are credible. Data are collected by trained local research staff using digital case report forms (CRFs), stored safely and encoded according to the central Information Technology (IT)-regime and standard operating procedures for FAIR (Findable, Accessible, Interoperable and Reusable) research data management. Questionnaires will be answered online and output will be stored in SPSS (Statistical Package for the Social Sciences). Raw data including metadata are available through a repository and under conditions available upon request.

### Confidentiality {27}

To ensure the privacy of participants, all the participants’ data will be encoded and only accessible to the principal investigator, coordinating investigator and project leader. Collected data from all centres will be stored at the Amsterdam UMC for 15 years.

### Plans for collection, laboratory evaluation, and storage of biological specimens for genetic or molecular analysis in this trial/future use {33}

Not applicable; these specimens are not collected.

## Statistical methods

### Statistical methods for primary and secondary outcomes {20a}

Data will be assessed using IBM SPSS Statistics Version 25. All statistical testing will be performed two-sided with *α* = .05 and according to the intention-to-treat-principle.

#### Primary study parameter

Primary outcome is the proportion of reoperations within 1 year after primary hernia repair. The number and percentage of reoperations by 1 year after primary hernia repair will be reported for each treatment group. Besides a crude analysis, a logistic regression analysis will be performed adjusted for centre and possible confounders (sex, gestational age at birth and initial hernia side). The primary-effect estimate will be the adjusted odds ratio, reported with the corresponding 95% confidence interval (CI).

#### Secondary study parameters

Secondary outcomes include the length of hospital stay and duration of operations (including anaesthesia time), complications of surgery and the health-related quality of life (HRQOL) of the operated infants and parental distress and anxiety. Mean (standard deviation, SD) or median (interquartile range, IQR) differences and corresponding 95% CIs will be calculated for length of hospital stay and duration of operations (including anaesthesia time). Odds ratio together with the 95% CI will be calculated for complications. Linear and logistic regression analysis will be performed to adjust for centre and possible confounders (sex, gestational age at birth and initial hernia side). Differences in HRQOL and parental distress and anxiety at baseline before surgery, 4 weeks and 1 year after primary hernia repair and, if relevant, prior to and 4 weeks after re-operation for MCIH will be reported using median and interquartile ranges.

#### Economic evaluation

The economic evaluation will be performed from both a societal and healthcare perspective in accordance with the intention-to-treat principle. The economic evaluation will be performed with the number of second operations and quality of life as outcomes. All relevant costs (costs within the healthcare system, costs for patient and family, and costs in other sectors) will be measured, valued and analysed. Healthcare costs are costs of the intervention (unilateral hernia repair with or without contralateral exploration) and costs of treatment of complications. Costs for patient and family include travel expenses, time spending costs and costs of informal care. Costs in other sectors include productivity loss due to work absenteeism by parents/caretakers. We will use a retrospective cost questionnaire and the iMTA Productivity Cost Questionnaire (iPCQ) to measure the direct and indirect costs; 4 weeks and 1 year after primary hernia repair and, if relevant, 4 weeks after re-operation. Costs will be valued using guideline prices recommended in the Netherlands Guideline for economic evaluations in healthcare (Netherlands Health Care Institute, Diemen, 2016)

For estimating quality-adjusted life years (QALY), the patients’ EQ-5D-5L health states (reported by parents/caretakers) will be converted into utility scores using the Dutch tariff [31]. QALYs will subsequently be calculated using linear interpolation between measurement points. Missing data will be imputed using multiple imputation by chained equations [32]. Incremental cost-effectiveness ratio (ICER) will be calculated by dividing the difference in costs by the difference in effects. A cost-effectiveness ratio is calculated to present the incremental costs per re-operation prevented. A cost-utility ratio expresses the incremental costs per QALY. In order to account for the possible clustering of data, analyses will be performed using linear multilevel analyses [33]. Bootstrapping techniques will be used to estimate the uncertainty surrounding the cost-effectiveness estimates. Uncertainty will be shown in cost-effectiveness planes and cost-effectiveness acceptability curves, and sensitivity analyses will be performed to test the robustness of the study results.

## Interim analyses {21b}

No interim analysis will be performed, as this can only be performed after the year follow-up, which will parallel the inclusion of all patients.

### Methods for additional analyses (e.g. subgroup analyses) {20b}

Pre-specified exploratory subgroup analyses will be performed on the primary outcomes stratified by sex (male versus female), gestational age (< 37 versus ≥ 37 weeks), initial hernia side (left versus right) and age at the time of surgery.

Parallel to this RCT, we will perform a qualitative study including a problem analysis for future implementation. We will use the framework of Fleuren et al. to explore facilitators and barriers for implementation of contralateral exploration during unilateral hernia repair on the level of the socio-political context, the organisation, healthcare professionals and the intervention itself [34]. Structured interviews will be held with all relevant stakeholders (e.g. parents, medical professionals, policy makers, patient organisations and health care insurance companies).

### Methods in analysis to handle protocol non-adherence and any statistical methods to handle missing data {20c}

Statistical tests will be performed according to an intention-to-treat principle. Efforts will be made in order to reduce missing data to a minimum. Missing data will be imputed using multiple imputation by chained equations. Mixed model analysis will be performed for longitudinal data.

### Plans to give access to the full protocol, participant level-data and statistical code {31c}

The trial protocol and statistical analysis plan will be made available via ‘figshare’. Completely deidentified datasets will be delivered to an appropriate data archive for sharing purposes and can be made available upon reasonable request and in agreement with our research collaboration and data transfer guidelines as stated in the Datamanagement plan.

## Oversight and monitoring

### Composition of the coordinating centre and trial steering committee {5d}

The HERNIIA trial is a multicentre study designed and coordinated in the Amsterdam UMC.

Principal investigator: responsible for all clinical research activities, thereby ensuring the study is conducted in accordance to the SOP and GCP guidelines.

Steering committee (see title page for members): assistance with the design of the study, agreement of final protocol and statistical analysis plan, reviewing progress of study and if necessary agreeing changes to the protocol.

Trial management committee (principal investigator, study coordinator and project leader): design and conduct of the trial, preparation of protocol and revisions, building the CRFs, study planning, trial registration, organisation of steering committee meetings, provide annual reports to ethics committees, (serious) adverse event ((S)AE) reporting, responsible for trial master file and site master files, contractual issues with participating centres, coordinating site visits, assistance with international review, board/independent ethics committee applications, data entry and verification, randomisation, publication of study reports, ensuring follow-up according to the protocol.

Lead investigators: in each participating centre a lead investigator is responsible for identification, recruitment, informed consent, data collection and completion of CRFs, along with follow-up of study participants and adherence to study protocol. They also assist in reviewing the progress of study and if necessary agreeing changes to the protocol.

### Composition of the data monitoring committee, its role and reporting structure {21a}

Both treatment strategies (unilateral hernia repair with contralateral exploration and unilateral hernia repair only) are currently performed in infants with unilateral inguinal hernia who need to undergo unilateral hernia repair. Consequently, there are no additional risks for subjects of this study and it is therefore not necessary to install a Data Safety Monitoring Board. Since this study is undertaken in infants and in order to make sure that the potential risks and burden of contralateral exploration does not outweigh the potential benefits, we installed an independent safety committee, that will provide assurance that the rights, safety and well-being of trial subjects are protected, and will monitor all complications and judge how to continue if a (S)AE is encountered.

### Adverse event reporting and harms {22}

All adverse events reported spontaneously by parents of the subject or observed by the investigator or his staff will be recorded from the start of the study until the moment of first follow-up. If a life-threatening SAE likely related to the study is encountered, this SAE will immediately be reported to the Central Committee on Research Involving Human Subjects (CCMO) according to the CCMO guidelines, and the study will be paused instantly, if appropriate. Inguinal hernia repair is usually at most accompanied by minimal complications.

### Frequency and plans for auditing trial conduct {23}

VU University Medical Centre provides an independent monitor who will perform interim monitoring. The monitor will verify that the rights and well-being of patients are protected, the reported trial data are accurate, complete and verifiable from source documents and the conduct of the trial is in compliance with the currently approved protocol/amendment(s), with GCP and with the applicable regulatory requirement(s). For more detailed information, the monitoring plan can be consulted.

### Plans for communicating important protocol amendments to relevant parties (e.g. trial participants, ethical committees) {25}

Amendments are changes made to the research after a favourable opinion by the accredited medical ethical research committee has been given. All amendments will be notified to the ethical committee that gave a favourable opinion. In case amendments concern or affect participants, they will be informed about the changes and additional informed consent will be requested, when necessary.

### Dissemination plans {31a}

We aim to disseminate the results of our study as soon as possible. Paediatric surgeons are familiar with both treatment strategies, ensuring optimal opportunities for implementation of the study results. If contralateral exploration is proved to be the best treatment strategy, we will endeavour rapid dissemination and implementation throughout the Netherlands, and potentially abroad.

Implementation activities of the findings entail understandable dissemination of results to parents via newsletters, patient information brochures and social media, presenting our results at (inter-)national conferences and publication in high-impact peer-reviewed international journals. Cost-effectiveness and potential scenarios for reimbursement is discussed with health insurers, Zorgverzekeraars Nederland (ZN) and Dutch Healthcare Authority (NZa).

## Discussion

The HERNIIA trial is the first randomised controlled trial that studies which treatment strategy is more (cost-)effective in infants with unilateral inguinal hernia: unilateral hernia repair with contralateral exploration or unilateral repair alone.

The “Child & Hospital Foundation” (a patient organisation devoted to child medical care; Dutch: “Stichting Kind & Ziekenhuis”, K&Z) and parents of infants with inguinal hernia are closely involved during the project to ensure optimal patient care. From a patient perspective, parents (interviewed at our clinic) and K&Z consider this study highly relevant and provided advice on the study design, patient information, patient reported outcomes and measurement techniques. They will be involved in every phase throughout the study for progress evaluation, advice on unexpected events and compiling content of dissemination and implementation activities for patients and parents after data analysis. The burden of the intervention will be assessed in panel discussions with parents/caretakers, by collecting information about adverse events and distribution of questionnaires. If relevant, K&Z (together with parents/caretakers) will support in developing a decision support tool to enable proper discussion of the results of the study with parents as part of shared-decision making.

## Trial status

Recruitment of participants started at 17 April 2019 and will be continued until the required number of participants will be enrolled. The current protocol is v.1.8, date 30 June 2020. We expect to complete the inclusion of patients in March 2022.

## Abbreviations

HERNIIA: Hernia Exploration oR Not In Infants Analysis
MCIH: Metachronous contralateral inguinal hernia
CCMO: Central Committee on Research Involving Human Subjects
PPV: Patent processus vaginalis
FDA: US Food and Drug Administration
RCT: Randomised controlled trial
SPIRIT: Standard Protocol Items: Recommendations for Interventional Trials
SOP: Standard operating procedure
GCP: Good clinical practices
WMO: Medical Research Involving Human Subjects Act
CPAP: Continues positive airway pressure
HRQOL: Health-related quality of life
TAPQOL: TNO-AZL Preschool Children Quality of Life
STAI: State-Trait Anxiety Inventory
EQ-5D-5L: EuroQol 5D
NICU: Neonatal intensive care unit
Castor EDC: Castor Electronic Data Capture
PROM: Patient-reported outcome measures
IT: Information Technology
SPSS: Statistical Package for the Social Sciences
CI: Confidence interval
SD: Standard deviation
IQR: Interquartile range
QALY: Quality-adjusted life years
ICER: Incremental cost-effectiveness ratio
SAE: Serious adverse event
ZN: Zorgverzekeraars Nederland
NZa: Dutch Healthcare Authority
K&Z: Child and Hospital Foundation
